# Correction: Effects of deterioration and mildewing on the quality of wheat seeds with different moisture contents during storage

**DOI:** 10.1039/d0ra90060e

**Published:** 2020-05-19

**Authors:** Ruolan Wang, Lulu Liu, Yapeng Guo, Xin He, Qian Lu

**Affiliations:** School of Food Science and Technology, Henan University of Technology Zhengzhou 450001 China wangruol@163.com qianlu1960@foxmail.com; School of Resources, Environmental & Chemical Engineering, Key Laboratory of Poyang Lake Environment and Resource Utilization, Ministry of Education, Nanchang University Nanchang 330031 China

## Abstract

Correction for ‘Effects of deterioration and mildewing on the quality of wheat seeds with different moisture contents during storage’ by Ruolan Wang *et al.*, *RSC Adv.*, 2020, **10**, 14581–14594, DOI: 10.1039/D0RA00542H.

The authors regret the omission of the e-mail address for one of the corresponding authors, Qian Lu, from the original manuscript. The corrected contact details are shown in this article.

The Royal Society of Chemistry apologises for these errors and any consequent inconvenience to authors and readers.

## Supplementary Material

